# Microtubules regulate tissue-level navigation in skin-resident macrophages

**DOI:** 10.1242/jcs.264101

**Published:** 2025-09-18

**Authors:** Eric Peterman, Andrew Murphy, Ian A. Swinburne, Tor H. Linbo, Sean G. Megason, Jeffrey P. Rasmussen

**Affiliations:** ^1^ Department of Biology, University of Washington, Seattle, WA 98195, USA; ^2^ Department of Systems Biology, Harvard Medical School, Boston, MA 02115, USA; ^3^ Department of Molecular Cell Biology, University of California, Berkeley, CA 94720, USA; ^4^ Department of Neurobiology and Biophysics, University of Washington, Seattle, WA 98195, USA; ^5^ Department of Otolaryngology-Head and Neck Surgery, University of Washington, Seattle, WA 98195, USA; ^6^ Institute for Stem Cell and Regenerative Medicine, University of Washington, Seattle, WA 98109, USA

**Keywords:** Microtubule, Tissue-resident macrophage, Migration, Phagocytosis, Skin, Zebrafish

## Abstract

Immune cells rapidly respond to tissue damage through dynamic properties of the cytoskeleton. How microtubules control immune cell functions during injury responses remains poorly understood. Within skin, tissue-resident macrophages known as Langerhans cells use dynamic dendrites to surveil the epidermis for damage and migrate through a densely packed epithelium to wounds. Here, we used Langerhans cells within the adult zebrafish epidermis as a model to investigate roles of microtubules in immune cell tissue surveillance, phagocytosis and directed migration. We describe microtubule organization within Langerhans cells and show that depolymerizing the microtubule cytoskeleton alters dendrite morphology, debris engulfment and migration efficiency. We found that the microtubule organizing center positions adjacent to engulfed debris and that its position correlates with navigational pathfinding during tissue-level migration. Stabilizing microtubules inhibits Langerhans cell motility during directed cell migration by impairing navigation around cellular obstacles. Collectively, our work demonstrates requirements for microtubules in the dynamic actions of tissue-resident macrophages during epithelial surveillance and wound repair.

## Teaser

**Highlighted Article:** Tissue-resident macrophages require microtubule dynamics to avoid epithelial obstacles during navigational pathfinding toward sites of tissue damage.

## INTRODUCTION

Immune cells must respond to wounds quickly to prevent pathogen invasion, clear debris and promote tissue repair. Rapid responses require that immune cells navigate complex, 3D tissue environments that can range from loosely organized interstitial spaces to densely packed epithelia (Delgado and Lennon-Duménil, 2022; Yamada and Sixt, 2019). Although studies from several systems have begun to reveal mechanisms of interstitial migration (Barros-Becker et al., 2017; Fernandes et al., 2020; Harada et al., 2012; Lämmermann et al., 2009; Nombela-Arrieta et al., 2007; Park et al., 2010; Tabdanov et al., 2021; Weber et al., 2013; Wilson et al., 2013; Yoo et al., 2010), navigation of immune cells within epithelia remains relatively understudied (Alraies et al., 2024; Renkawitz et al., 2019). Understanding how immune cells migrate through solid tissues is relevant to studies of diverse organ and tumor microenvironments.

The superficial location of skin makes it an excellent model for studying epithelial biology. The outermost layer of skin, the epidermis, is an epithelium composed of tightly adherent layers of keratinocytes that form a watertight and mechanical barrier. Various resident immune cells are interspersed among and confined by epidermal keratinocytes, complicating their navigation in this dense microenvironment (Nestle et al., 2009). Upon skin damage, resident cells quickly mobilize to restore organ function: keratinocytes rearrange to restore the physical barrier, while immune cells migrate to the wound to clear cell debris (Peña and Martin, 2024). We and others recently found that tissue-resident macrophages known as Langerhans cells migrate to sites of damage and promote skin repair (Lin et al., 2019; Peterman et al., 2024; Wang et al., 2024; Wasko et al., 2022). Langerhans cells populate the epidermis and other epithelial tissues, such as oral and vaginal mucosa, surveilling tissue and acting as multimodal immune cells capable of emigration to lymph nodes and responding to tissue damage (Brand et al., 2023; Larsen et al., 1990; Lin et al., 2019; Peterman et al., 2024; Vine et al., 2024; Wang et al., 1999a,b, 2024; Wasko et al., 2022). The rapid migration of Langerhans cells to sites of tissue damage makes them an attractive model to study immune cell navigation, but how they pathfind through the confined epidermal microenvironment remains unknown.

Langerhans cells adopt a ramified morphology in homeostatic skin, and their long and dynamic dendrites facilitate skin surveillance and cell spacing (Kubo et al., 2009; Nishibu et al., 2006; Park et al., 2021). Upon skin damage, Langerhans cells modulate their dendritic morphology to accommodate cell engulfment (Peterman et al., 2024). Arborized and ramified cells such as neurons and microglia, respectively, rely on the actin and microtubule cytoskeletal networks to promote cell functionality. Work by our group and others suggest that actin and actin-regulating networks control Langerhans cell spacing, dendrite dynamics and migration in response to tissue damage (Park et al., 2021; Peterman et al., 2024). Microtubules and the microtubule organizing center (MTOC) function prominently during immune cell migration through interstitial microenvironments (Renkawitz et al., 2019; Yoo et al., 2012). Previous studies implicate microtubules in Langerhans cell antigen uptake and presentation (Bacci et al., 1996; Martin et al., 2014), but how microtubules function during immune cell migration through solid epithelia remains an open question.

**Fig. 1.**
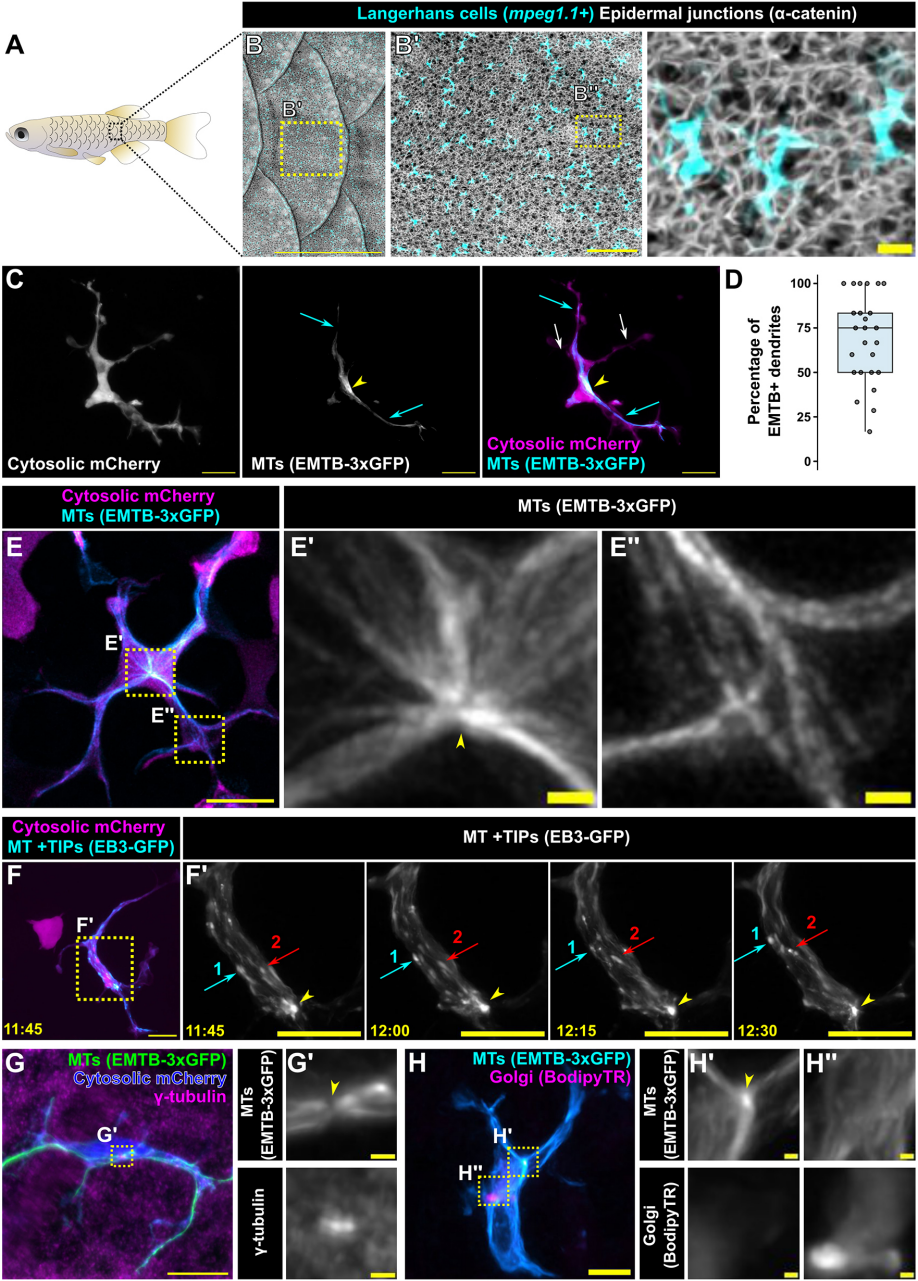
**Langerhans cells possess a single perinuclear microtubule organizing center.** (A) Illustration of adult zebrafish showing overlapping scales along the trunk skin. (B–B″) Confocal images of adult trunk skin expressing reporters for Langerhans cells [*Tg(mpeg1.1:mCherry)*] and α-catenin [*Gt(ctnna1-Citrine)*], which labels epidermal junctions (Trinh et al., 2011). Images are representative of three experimental repeats. (C) Representative image of a Langerhans cell expressing *Tg(mpeg1.1:mCherry;mpeg1.1:EMTB-3xGFP)*, showing EMTB-3xGFP^+^ dendrites (cyan arrows), EMTB-3xGFP^−^ dendrites (white arrows) and the presumptive microtubule organizing center (MTOC) (yellow arrowheads). (D) Box plot showing percentage of EMTB^+^ dendrites (*n=*25 cells from 12 scales). Whiskers extend to minimum and maximum values; boxed lines represent quartile ranges and median. (E–E″) Super-resolution microscopy of a Langerhans cell expressing *Tg(mpeg1.1:mCherry;mpeg1.1:EMTB-3xGFP)*, showing a single perinuclear MTOC (E′, yellow arrowhead) and microtubules in dendrites (E″). Images are representative of one experimental repeat. (F,F′) Representative still images from time-lapse confocal microscopy of a *Tg(mpeg1.1:mCherry;mpeg1.1:EB3-GFP)^+^* Langerhans cell showing the perinuclear focus of EB3 signal (yellow arrowheads). Yellow dotted line box in left panel denotes region magnified in the panels on the right. Red and cyan arrows track individual EB3-GFP^+^ puncta. Images are representative of two experimental repeats. (G,G′) Representative images of a fixed Langerhans cell expressing *Tg(mpeg1.1:mCherry;mpeg1.1:EMTB-3xGFP)* stained with an anti-γ-tubulin antibody. Yellow dotted line box in G denotes the region magnified in G′. Yellow arrowhead in G′ indicates the MTOC. Images are representative of three experimental repeats. (H–H″) Representative images of a live Langerhans cell expressing *Tg(mpeg1.1:EMTB-3xGFP)* stained with BodipyTR. Yellow dotted line boxes in H denote regions magnified in H′,H″. Yellow arrowhead in H′ indicates the MTOC. Timestamps denote min:s. Images are representative of one experimental repeat. Scale bars: 1 mm (B), 100 μm (B′), 10 μm (B″,C,E,F,F′,G,H), 1 μm (E′,E″,G′,H′,H″).

Owing to its optical clarity, zebrafish skin is an excellent model for studying immune cell motility in a native microenvironment. Along the fish trunk, the epidermis drapes across plate-like scales, a type of dermal appendage (Fig. 1A,B) (Aman and Parichy, 2024). Similarly to other types of vertebrate skin, the adult zebrafish epidermis contains stratified keratinocytes and interspersed immune cells, including T cells and Langerhans cells (Fig. 1B) (Dee et al., 2016; He et al., 2018; Hui et al., 2017; Lin et al., 2019; Robertson et al., 2023). Previously, we developed an *ex vivo* scale explant model that allows the visualization of Langerhans cell behaviors with high spatiotemporal resolution and acute cytoskeletal manipulation via chemical perturbations (Guerrero et al., 2024; Peterman et al., 2023, 2024). Here, we use this skin explant system in combination with new transgenes to examine the organization and function of microtubules and the MTOC during Langerhans cell skin surveillance and responses to tissue damage.

## RESULTS

### Microtubule organization in Langerhans cells in homeostatic skin

The zebrafish *macrophage expressed gene 1.1* (*mpeg1.1*) promoter labels diverse macrophage populations, including Langerhans cells (Ellett et al., 2011; He et al., 2018; Lin et al., 2019; Zhou et al., 2023). To visualize microtubules in Langerhans cells, we created a transgenic line in which the *mpeg1.1* promoter drives expression of a microtubule reporter derived from the microtubule binding domain of Ensconsin fused to three copies of GFP (EMTB-3xGFP) [*Tg(mpeg1.1:EMTB-3xGFP)*] (Barros-Becker et al., 2017; Faire et al., 1999). To simultaneously visualize Langerhans cell microtubules and cytoplasm, we crossed this line to *Tg(mpeg1.1:mCherry)* (Ellett et al., 2011), which expresses cytosolic mCherry in Langerhans cells (Fig. 1C; Movie 1). From confocal *z*-stacks, we identified linear arrays of EMTB-3xGFP signal in at least one dendrite of all Langerhans cells examined (Fig. 1C, yellow arrowheads; Fig. 1D; range, 16.67–100% EMTB-3xGFP^+^ dendrites/cell; *n*=25 cells). Treatment of scale explants with nocodazole resulted in loss of EMTB-3xGFP signal (Fig. S1), indicating that our reporter labeled microtubules and that nocodazole depolymerized microtubules in our explant system.

Immune cells often possess radially organized microtubules that nucleate from a MTOC located at the centrosome or Golgi complex (Meiring et al., 2020). Previous studies have used EMTB^+^ foci to identify the MTOC of migrating dendritic cells (Kroll et al., 2023; Renkawitz et al., 2019). While imaging EMTB-3xGFP localization in Langerhans cells, we observed that microtubules appeared to arise from the brightest point of signal, which localized near the nucleus (Fig. 1C, yellow arrowheads), suggesting this represents the MTOC. To gain a higher-resolution view of microtubule structures, we performed super-resolution microscopy and observed microtubule arrays emanating from this bright perinuclear focus (Fig. 1E,E′). Although Langerhans cell dendrites contained intricate microtubule organization, they lacked microtubule foci at branch points (Fig. 1E″). As a complementary approach to MTOC identification, we used the plus end (+TIP)-localized End-binding protein 3 (EB3) (Stepanova et al., 2003) to label the growing +TIPs of microtubules in Langerhans cells of *Tg(mpeg1.1:mCherry)* adults. We found that EB3-GFP^+^ puncta emerged from a bright, perinuclear EB3-GFP^+^ focus (Fig. 1F; Movie 2). To further describe the identity of the MTOC, we performed anti-γ-tubulin immunostaining to label the centrosome and BodipyTR staining to label the Golgi complex. The perinuclear concentration of EMTB signal colocalized with γ-tubulin but not BodipyTR (Fig. 1G,H). Based on these combined data, we concluded that Langerhans cell dendrites contain microtubules that nucleate from a single, perinuclear centrosomal MTOC.

### Microtubules maintain Langerhans cell morphology

**Fig. 2.**
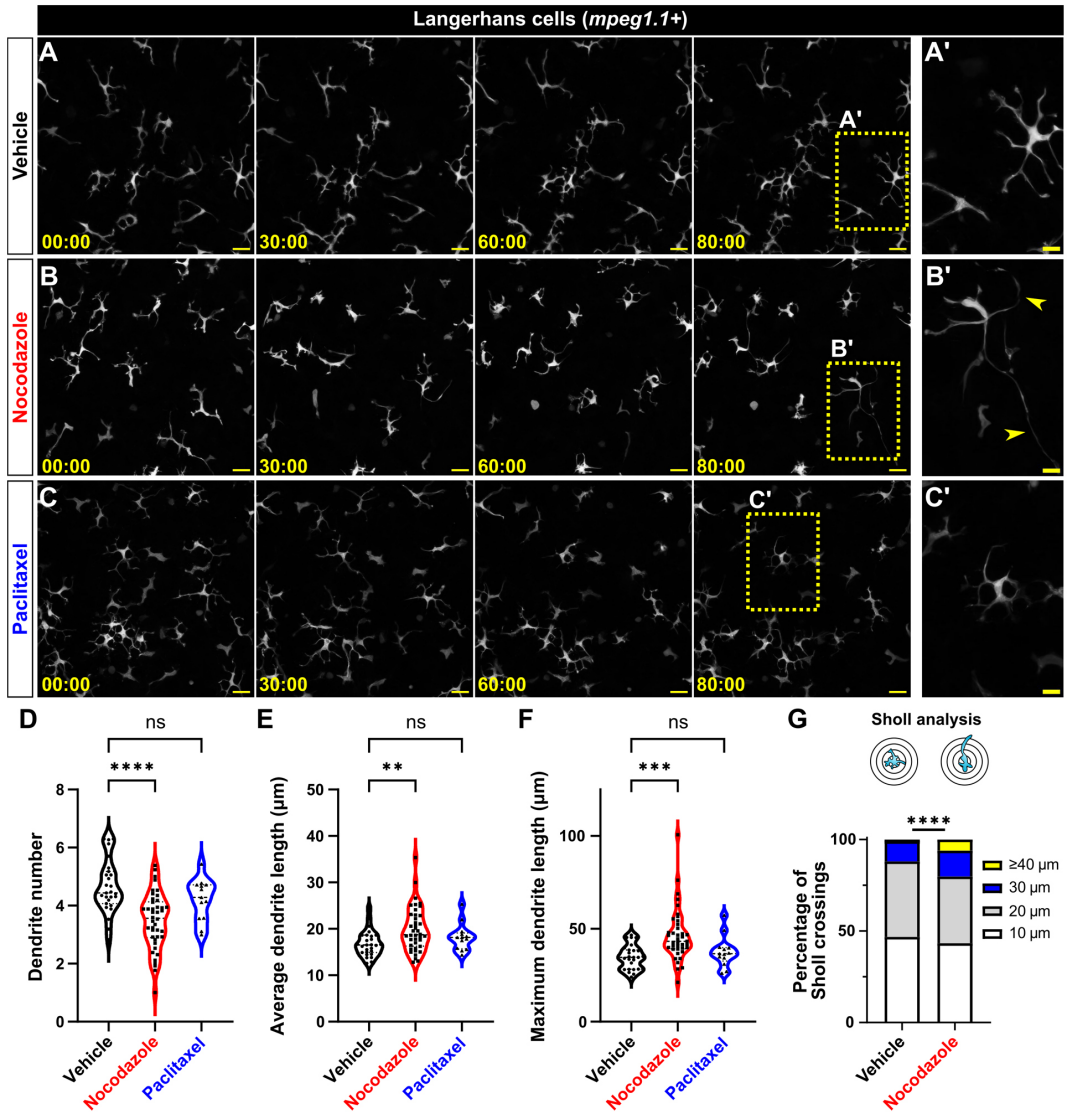
**Microtubule depolymerization disrupts Langerhans cell morphology.** (A–C′) Representative still images from time-lapse confocal microscopy depicting effects of vehicle (A), nocodazole (B) or paclitaxel (C) treatment on *Tg(mpeg1.1:YFP)*^+^ Langerhans cell morphology. Yellow arrowheads in B′ indicate elongated dendrites. (D–F) Violin plots showing average dendrite number (D), average dendrite length (E) or maximum dendrite length (F) through 80 min of vehicle, nocodazole or paclitaxel treatment. *n*=27 cells tracked from seven scales for vehicle control from three individual experiments, *n=*42 cells tracked from eight scales for nocodazole from five individual experiments, *n=*13 cells tracked from six scales for paclitaxel from four individual experiments. (G) Sholl analysis of dendrite lengths in vehicle- and nocodazole-treated conditions. *n=*698 dendrites tracked in 14 cells from three scales from three individual experiments for vehicle control and *n*=476 dendrites tracked in 11 cells from three scales from three individual experiments for nocodazole. Statistical significance in D–F was determined using Mann–Whitney *U*-test, statistical significance in G was determined using a Chi-squared test with the raw number of dendrites. ns, not significant; **P<0.01, ***P<0.001, ****P<0.0001. Timestamps denote min:s. Scale bars: 10 μm (A′,B′,C′), 20 μm (A,B,C).

To test whether maintenance of Langerhans cell morphology requires microtubules, we explanted skin and administered nocodazole to depolymerize microtubules (Fig. 2A,B; Movie 3). We analyzed Langerhans cell morphology using the cytosolic reporter *Tg(mpeg1.1:YFP)* (Roca and Ramakrishnan, 2013) and found significantly decreased dendrite number in nocodazole-treated cells compared to that in vehicle-treated controls (Fig. 2D). By contrast, nocodazole treatment significantly increased average and maximum dendrite lengths (Fig. 2E,F). As an alternative measure of dendrite morphology, we used Sholl analysis to measure the number of dendrites crossing concentric circles in increasing 10 μm intervals. Our analysis revealed that microtubule depolymerization significantly increased the range of dendrite lengths (Fig. 2G). To examine how microtubule stabilization affected Langerhans cell morphology, we also treated scale explants with paclitaxel, a microtubule-stabilizing drug (Schiff and Horwitz, 1980; Schiff et al., 1979). Treatment of scale explants with paclitaxel did not alter dendrite number or length (Fig. 2C–F). These data indicate that acute microtubule depolymerization, but not stabilization, alters Langerhans cell dendrite number and length in steady-state conditions.

### The MTOC rapidly localizes to sites of debris engulfment

**Fig. 3.**
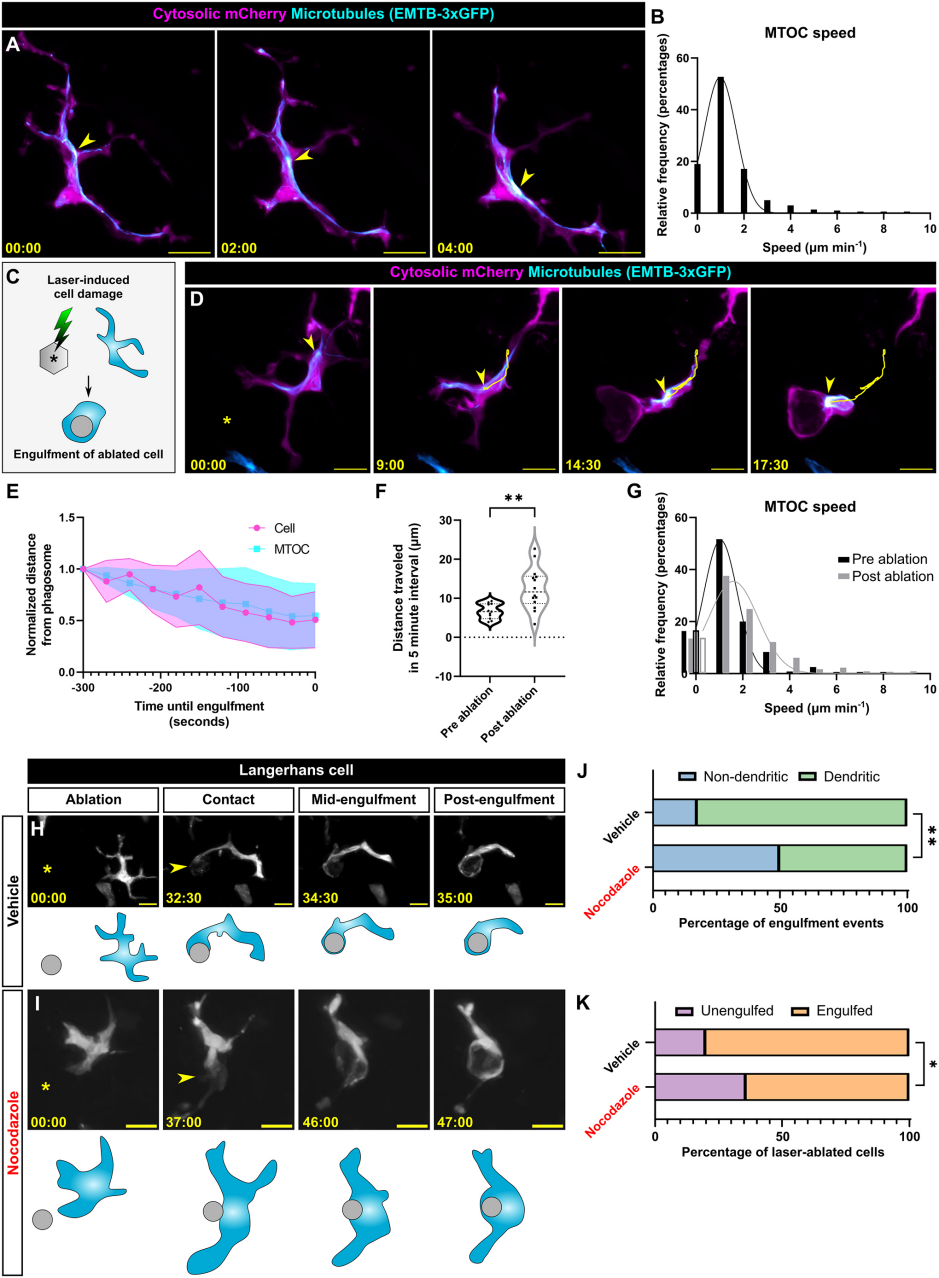
**MTOC dynamics and microtubule requirements during debris engulfment.** (A) Representative still images from time-lapse confocal microscopy of a *Tg(mpeg1.1:mCherry;mpeg1.1:EMTB-3xGFP)^+^* Langerhans cell showing MTOC (yellow arrowheads) movement in steady-state conditions. (B) Histogram showing the frequency distribution of MTOC speed in steady-state conditions, *n=*10 cells tracked from five scales. (C) Schematic of laser-induced keratinocyte (cyan) damage and subsequent Langerhans cell engulfment. (D) Representative still images from time-lapse confocal microscopy showing MTOC (yellow arrowheads) motility preceding engulfment of debris generated by keratinocyte laser ablation. Yellow trace indicates the MTOC track over time. Yellow asterisk indicates the site of laser ablation. (E) Quantification of the relative distance between the cell or MTOC and phagosome in the 5 min prior to debris engulfment, *n=*10 cells tracked from five scales. Shading represents standard deviation. (F) Violin plot of the total distance traveled by the MTOC 5 min prior to keratinocyte laser ablation and 5 min prior to keratinocyte engulfment, *n=*11 cells tracked in pre ablation, *n=*14 cells tracked in post ablation from seven scales. (G) Histogram of the frequency distribution of MTOC speed 5 min prior to keratinocyte laser ablation and 5 min prior to keratinocyte engulfment, *n=*6 cells tracked in pre ablation, *n=*9 cells tracked in post ablation from four scales. (H,I) Representative still images from time-lapse confocal microscopy of vehicle-treated (H) or nocodazole-treated (I) *Tg(mpeg1.1:YFP)^+^* Langerhans cells showing engulfment of debris. Yellow asterisks indicate the site of laser ablation. Yellow arrowheads indicate the site of first contact between the Langerhans cell and laser-damaged cell. (J) Quantification of engulfment modality used to engulf debris, *n=*40 engulfment events tracked from four experiments for vehicle control, *n=*34 ablation events tracked from four experiments for nocodazole. (K) Quantification of successful keratinocyte debris engulfment following addition of vehicle or nocodazole, *n=*50 ablated cells tracked from four experiments for vehicle control, *n=*53 ablated cells tracked from four experiments for nocodazole. Statistical significance in F was determined using a Mann–Whitney *U*-test. A Kolmogorov–Smirnov test was used in G and revealed no significant difference. Fisher's exact test was used to determine significance in J,K by using the raw counts of engulfment events. Traces in frequency distribution graphs B and G are Gaussian fits. *P<0.05, **P<0.01. Timestamps denote min:s. Scale bars: 10 μm.

The microtubule cytoskeleton and MTOC can rapidly reorient in response to external stimuli, such as during the formation of the immunological synapse by T cells and phagocytosis by microglia (Möller et al., 2022; Yi et al., 2013). To analyze MTOC dynamics in Langerhans cells, we began by measuring MTOC motility during steady-state conditions and observed that the brightest EMTB-3xGFP focus per cell (hereafter referred to as the MTOC) moved an average of 1.4 μm min^−1^ in the absence of stimulus (Fig. 3A,B; Movie 1). Next, we tracked Langerhans cell engulfment of laser-damaged single keratinocytes as previously described (Fig. 3C) (Guerrero et al., 2024; Peterman et al., 2024). Consistent with observations in microglia (Möller et al., 2022), we found that the Langerhans cell MTOC relocalized to the site of debris contact after laser ablation (Fig. 3D,E; Movie 4). However, unlike previous work (Möller et al., 2022), MTOC motility in Langerhans cells closely corresponded with cell body motility, suggesting that the cell and MTOC move in concert rather than due to independent MTOC motility. When we quantified the distance that the MTOC traveled 5 min prior to ablation and 5 min prior to engulfment, we found that the MTOC traveled further distances following Langerhans cell activation (Fig. 3F). Additionally, we found that MTOC speed was faster after laser ablation (average of 1.8 μm min^−1^) compared to that pre-ablation (average of 1.3 μm min^−1^) (Fig. 3G). Altogether, these data show that Langerhans cell MTOC motility increases after nearby cellular damage and that the MTOC relocalizes proximal to debris during engulfment.

### Dendrite-mediated engulfment of debris requires the microtubule cytoskeleton

Our observations that (1) microtubule depolymerization alters Langerhans cell dendrite morphology and that (2) the MTOC polarizes towards cell debris after laser ablation led us to hypothesize that microtubules are required for debris engulfment. To test this, we used our laser ablation paradigm in combination with vehicle or nocodazole treatment and tracked debris engulfment. In vehicle-treated conditions, we observed two modes of debris engulfment by Langerhans cells. The predominant modality involved Langerhans cells contacting debris with a single, long dendrite, while simultaneously retracting distal dendrites prior to debris engulfment (Fig. 3H,J; 82.5% of events; Movie 5). Alternatively, Langerhans cells adopted a non-dendritic modality in which the entire cell migrated and repositioned itself next to the debris, which was then engulfed at the cell body (Fig. 3J; 17.5% of events). In nocodazole-treated conditions, Langerhans cells exhibited increased usage of the motility-based engulfment modality (Fig. 3I,J) and a decrease in the ability to engulf debris (Fig. 3K). These results suggest that microtubules are partly responsible for dendrite-mediated engulfment and the overall engulfment success of damaged keratinocytes.

### Efficient directed cell migration requires the microtubule cytoskeleton

**Fig. 4.**
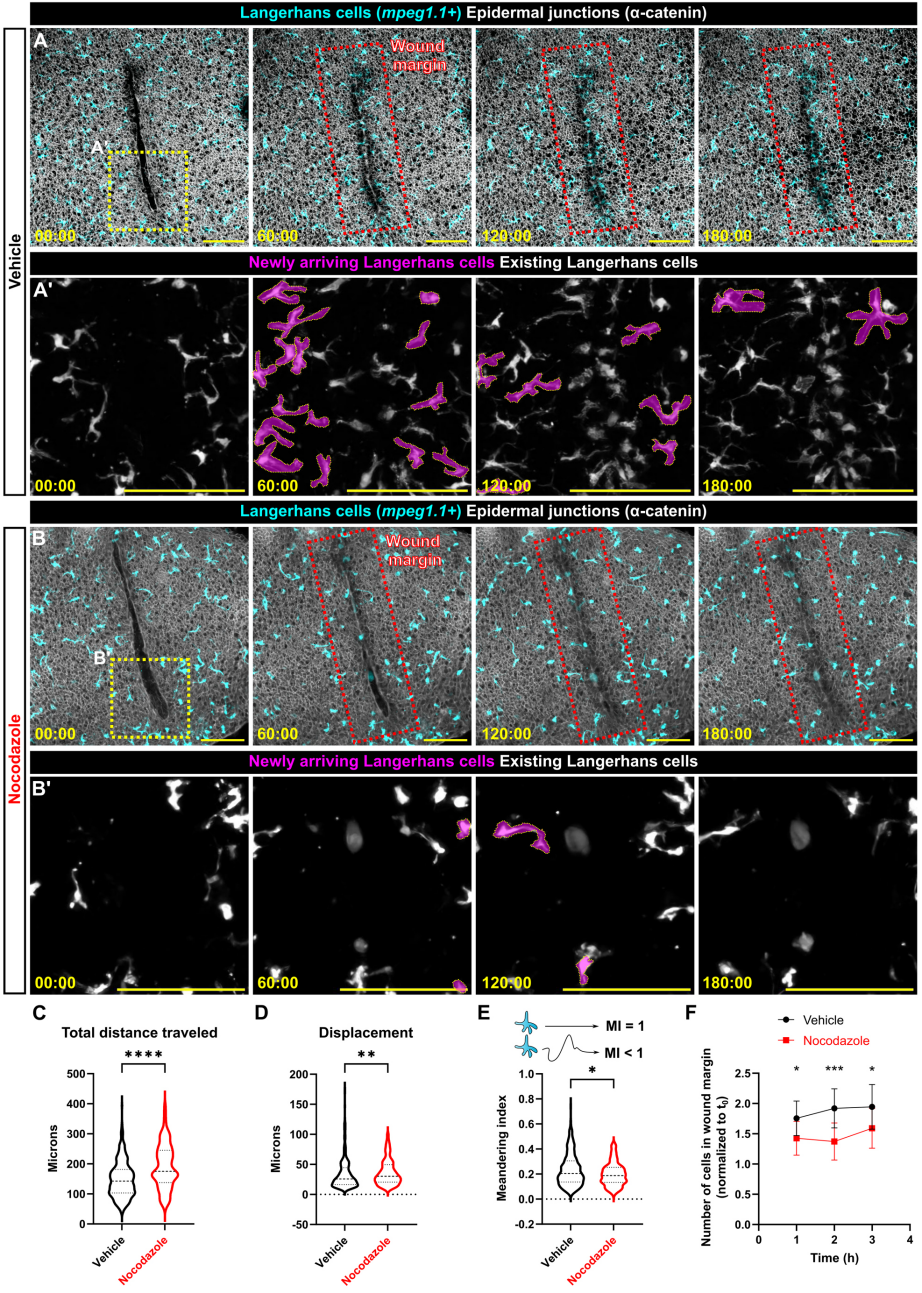
**Langerhans cell migration to scratch wounds requires microtubules.** (A,B) Langerhans cell migration to epidermal scratch in vehicle-treated (A) or nocodazole-treated (B) *Tg(mpeg1.1:mCherry)*;*Gt(ctnna1-Citrine)* skin explant. Yellow dotted line boxes in first frames of A,B indicate insets in A′,B′, respectively; red dotted line boxes in frames 2–4 indicate the wound margin region of interest corresponding to cell counts in F. Newly arriving Langerhans cells are pseudocolored magenta in A′,B′. (C–E) Violin plots showing total distance traveled (C), displacement (D) or the meandering index (MI; E) of *mpeg1^+^* cells over 3 h in response to epidermal scratch in vehicle- or nocodazole-treated conditions, *n=*325 cells from 11 skin explants from three experiments tracked in vehicle conditions, *n=*181 cells from ten skin explants from four experiments tracked in nocodazole conditions. (F) Quantification of the normalized number of *mpeg1^+^* cells in the wound margin in vehicle- or nocodazole-treated conditions, *n*=13 skin explants from three experiments for vehicle conditions, *n*=12 skin explants from four experiments for nocodazole conditions. Dots represent averages, error bars represent s.d. Mann–Whitney *U*-tests were used to determine significance in C–E, two-way ANOVA followed by Bonferroni post-test was used to determine significance in F. *P<0.05, **P<0.01, ***P<0.001, ****P<0.0001. Timestamps denote min:s post-scratch. Scale bars: 100 μm.

In addition to their function in regulating phagocytosis, microtubules and the MTOC play pivotal roles in immune cell migration (Etienne-Manneville, 2013). Previous work demonstrated that murine and zebrafish Langerhans cells migrate to tissue wounds generated by punch biopsy or mechanical scratch, respectively (Lin et al., 2019; Peterman et al., 2024; Wasko et al., 2022). However, the underlying mechanisms that promote Langerhans cell migration remain poorly understood. To ask whether Langerhans cells require microtubules for directional migration, we pretreated explanted scales with vehicle or nocodazole, introduced an epidermal scratch and imaged cell migration over 3 h (Fig. 4A,B; Movie 6). Compared to vehicle-treated controls, nocodazole-treated cells migrated with a significantly larger total distance traveled and displacement (Fig. 4C,D). As a measure of the efficiency of migration, we calculated the meandering index (defined as the ratio of displacement to total distance traveled) and found that Langerhans cells had a smaller meandering index, indicating that the cells did not migrate as directly toward the wound (Fig. 4E). Confirming this, we observed significantly fewer nocodazole-treated cells in the wound margin (Fig. 4F). As a control, we measured wound closure rates in vehicle- or nocodazole-treated scales and found that wounds closed at equal rates, suggesting that microtubule perturbation is not indirectly altering Langerhans cell migration through effects on wound closure (Fig. S2). Taken together, these data indicate that microtubule depolymerization causes increased Langerhans cell motility but with less directionality, leading to reduced cell accumulation at sites of tissue damage.

### Microtubule depolymerization alters actin distribution in migrating Langerhans cells

**Fig. 5.**
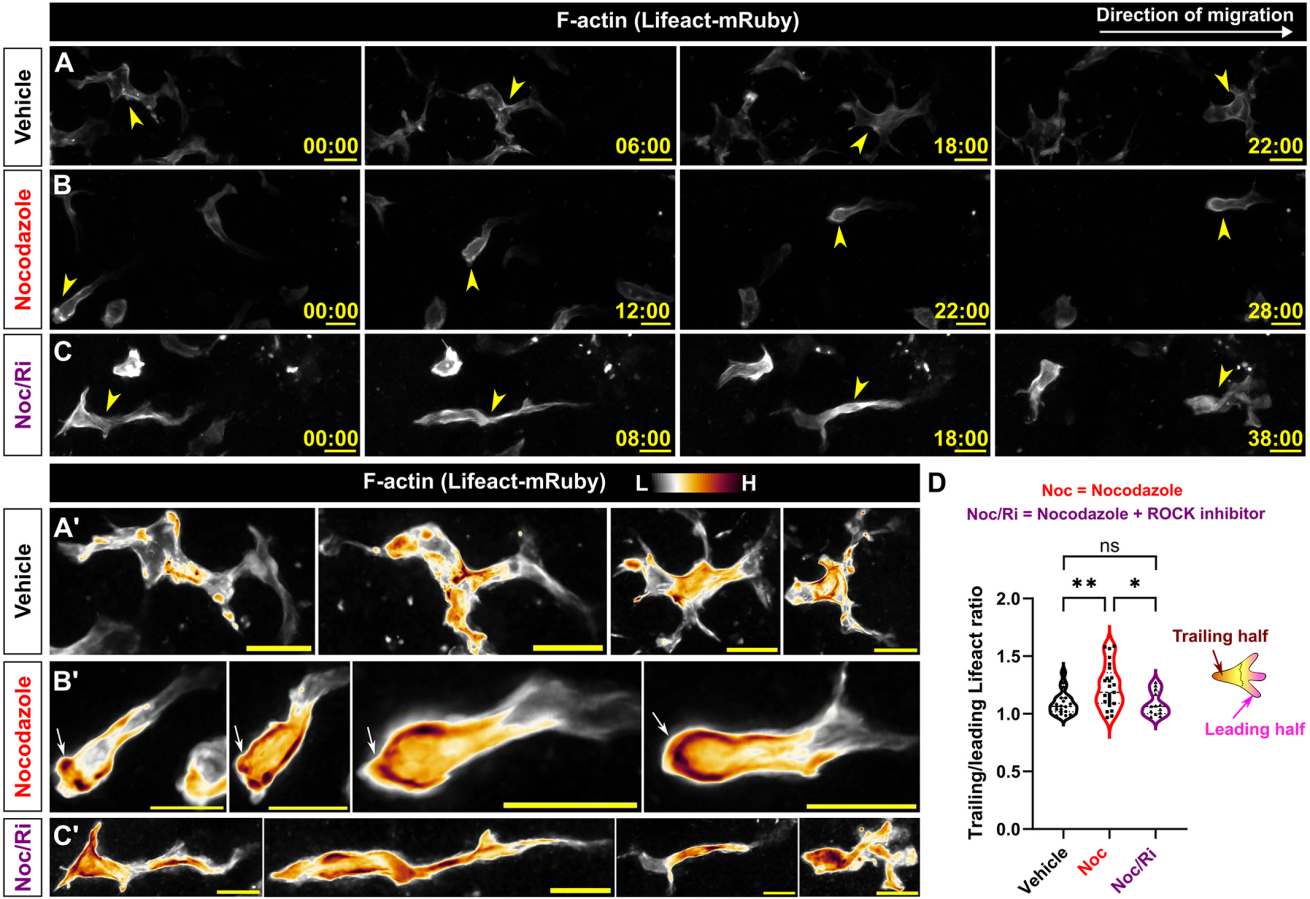
**Microtubule depolymerization increases F-actin levels in a ROCK-dependent manner in the trailing halves of migrating Langerhans cells.** (A–C′) Stills from time-lapse confocal microscopy showing migrating *Tg(mpeg1.1:Lifeact-mRuby)^+^* Langerhans cells treated with vehicle (A,A′), nocodazole (B,B′) or nocodazole+Y-27632 (Noc/Ri) (C,C′). Magnified insets in A′,B′,C′ are false colored to show Lifeact-mRuby levels. Arrowheads in A,B,C indicate migrating cell, arrows in B′ indicate increased Lifeact-mRuby levels in the trailing half of a nocodazole-treated cell. (D) Violin plot quantifying the ratio of Lifeact-mRuby at the trailing and leading halves (see Materials and Methods for analysis detail), *n*=20 cells tracked from three experiments in vehicle conditions, *n*=25 cells tracked from four experiments in nocodazole-treated conditions, *n*=13 cells tracked from six experiments in nocodazole+ROCK inhibitor (Y-27632)-treated conditions. Significance was determined using one-way ANOVA followed by Bonferroni post-test. ns, not significant; *P<0.05, **P<0.01. Timestamps denote min:s. Scale bars: 10 μm.

We next asked how microtubules promote efficient Langerhans cell migration to wounds. Previous works indicate multiple modes of crosstalk between the microtubule and actin cytoskeletons (Dogterom and Koenderink, 2019). One such mechanism involves sequestration of the RhoA activator guanine nucleotide exchange factor-H1 [GEF-H1, also known as Rho/Rac guanine nucleotide exchange factor 2 (ARHGEF2)] through binding to microtubules (Heck et al., 2012; Kashyap et al., 2019; Krendel et al., 2002). Microtubule depolymerization releases GEF-H1 from the microtubule lattice, leading to RhoA activation and subsequent changes in cell shape and actin distribution. Notably, our previous work suggests that a RhoA/Rho-associated kinase (ROCK)–myosin pathway promotes Langerhans cell migration to tissue wounds (Peterman et al., 2024). We hypothesized that Langerhans cell microtubule depolymerization alters RhoA activity, thereby disrupting actin distribution during migration towards wounds. To visualize F-actin in Langerhans cells, we used skin explants from *Tg(mpeg1.1:Lifeact-mRuby)* fish (Peterman et al., 2024). After explant, we pretreated scales with vehicle or nocodazole and scratched the epidermis. We followed individual cells as they migrated towards the wound and quantified Lifeact-mRuby ratios in the leading and trailing halves of migrating Langerhans cells. In vehicle-treated cells, we found roughly equal amounts of Lifeact-mRuby in the leading and trailing halves (Fig. 5A,A′,D). By contrast, nocodazole-treated cells contained an enrichment of Lifeact-mRuby in the trailing half, consistent with the idea that depolymerizing microtubules increases RhoA activity at the rear of the cell (Fig. 5B, arrowheads; Fig. 5B′,D). To inhibit a prominent downstream target of RhoA, we next used the ROCK inhibitor Y-27632 (Ishizaki et al., 2000), which we previously used to modulate Langerhans cell damage responses (Peterman et al., 2024). The addition of Y-27632 to nocodazole-treated explants restored Lifeact-mRuby ratios to near-control levels (Fig. 5C,D). Owing to technical limitations, we were unable to visualize RhoA or GEF-H1 in Langerhans cells. Nevertheless, these data suggest that depolymerization of microtubules alters actin distribution through a ROCK-dependent mechanism in migrating cells, which is one plausible cause of altered cell migration.

### Pathfinding during directional cell migration involves MTOC decision-making

**Fig. 6.**
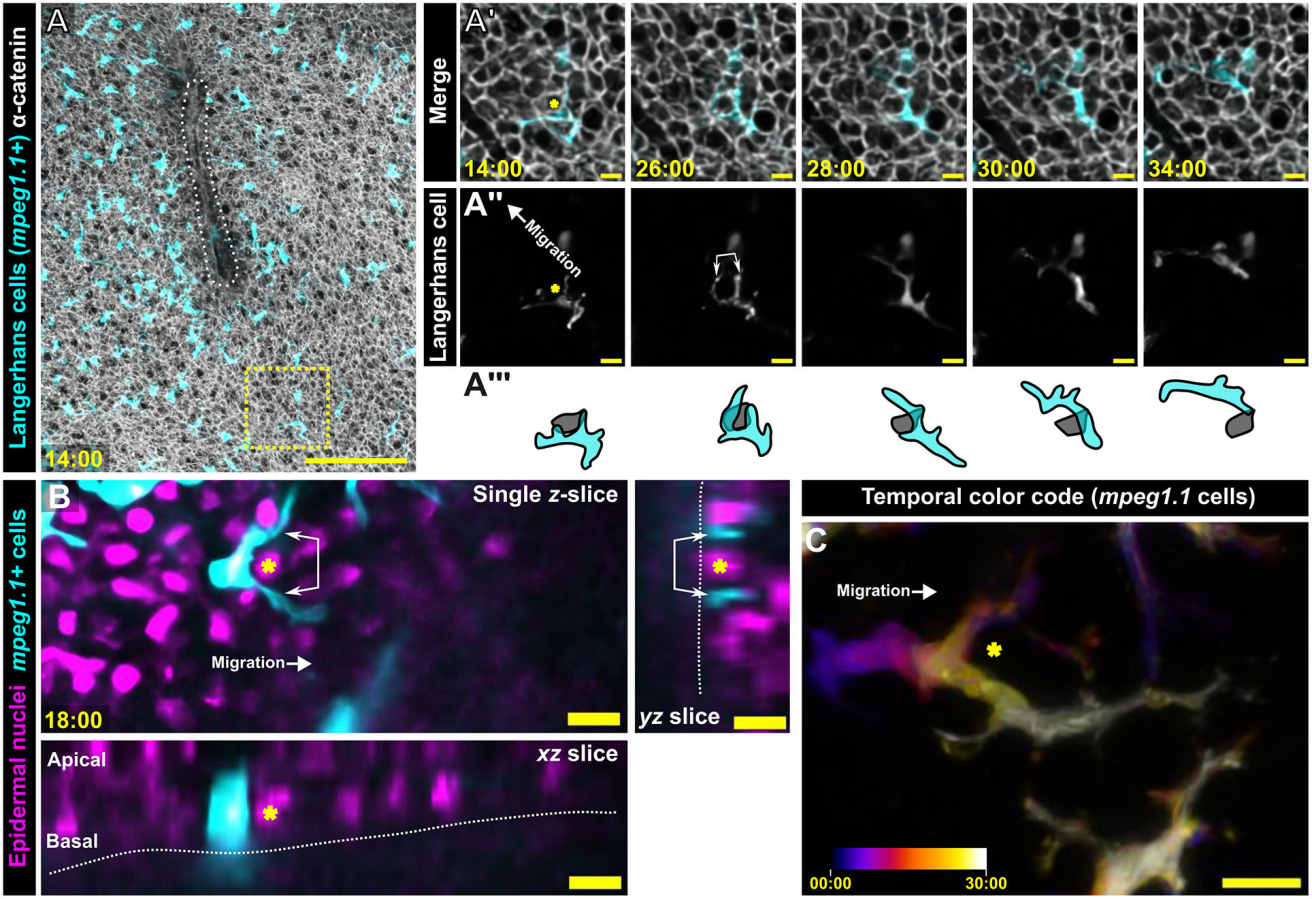
**Langerhans cells use dendrites to probe and navigate around epidermal cells en route to scratch wounds.** (A) Maximum-intensity projection from time-lapse confocal microscopy of *Tg(mpeg1.1:mCherry)*;*Gt(ctnna1-Citrine)* skin explant. White dotted outline indicates epidermal scratch. Yellow box in A is magnified in A′,A″ insets as single *z*-slices. Yellow asterisks indicate obstacle keratinocyte. Images are representative of 11 experimental repeats. (B) Still images from time-lapse microscopy of *Tg(mpeg1.1:YFP)*;*Tg(actb2:H2B-2x-mScarlet)* skin explant. Double-headed arrows indicate two Langerhans cell dendrites probing around an obstacle nucleus (yellow asterisks). White dotted outlines indicate the scale surface. Images are representative of four experimental repeats. (C) Temporal color coding of the *mpeg1.1^+^* cell from B navigating around an obstacle nucleus (yellow asterisk). Images are representative of four experimental repeats. Timestamp denote min:s post-scratch. Scale bars: 100 μm (A), 10 μm (A′–C).

While imaging Langerhans cell migration to epidermal wounds, we observed that Langerhans cell bodies would often transiently pause while their dendrites continued to extend and retract (Fig. 6A–A″). Close examination of this phenomenon revealed that keratinocytes appeared to impede Langerhans cell migration to the wound (Fig. 6A‴). To facilitate imaging nuclear position of all epidermal cells, we generated a transgenic line in which the quasi-ubiquitous *beta-actin2* (*actb2*) promoter (Kobayashi et al., 2014) drives expression of histone H2B fused to a tandem repeat of the red fluorescent protein mScarlet (Bindels et al., 2017) [*Tg(actb2:H2B-2x-mScarlet)*]. By scratching and imaging scale explants from *Tg(mpeg1.1:YFP;actb2:H2B-2x-mScarlet)* fish, we found that Langerhans cells extended their protrusions laterally beyond obstacle nuclei positioned in the basal epidermal layer (Fig. 6B) before ultimately passing the obstacle by choosing one side of the bifurcation (Fig. 6C). These data suggest that encounter with keratinocyte obstacles triggers a decision-making process within migrating Langerhans cells.

**Fig. 7.**
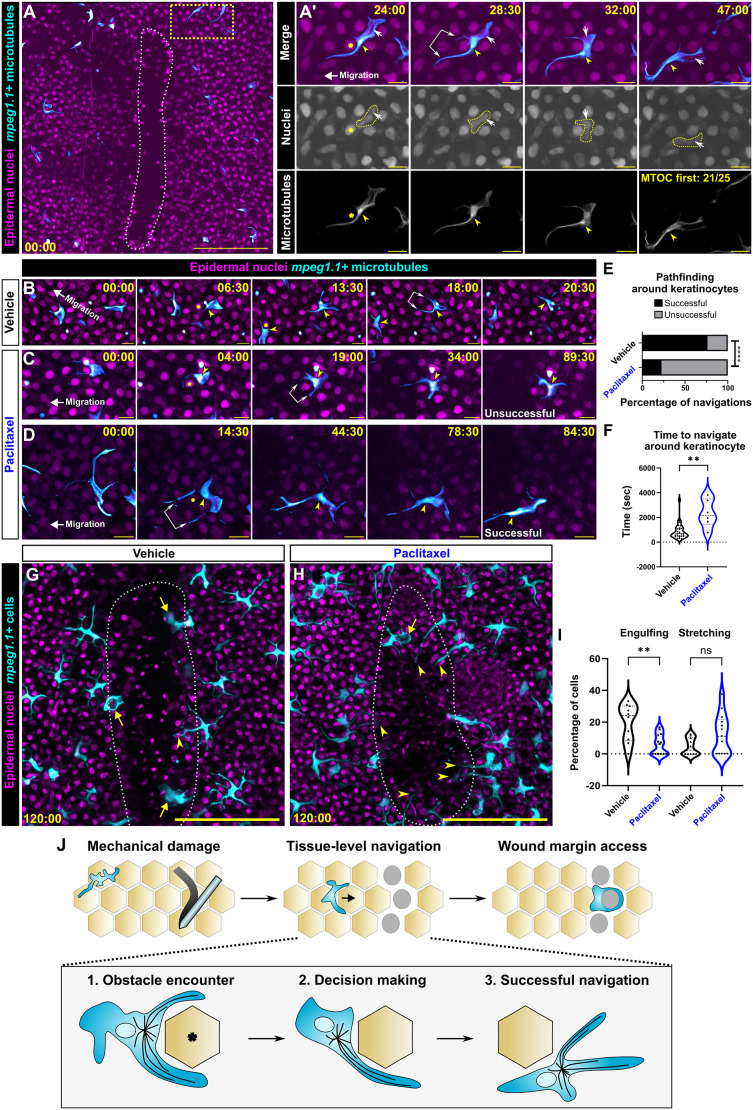
**MTOC motility correlates with navigational pathfinding towards large epidermal wounds.** (A) Representative still images from time-lapse confocal microscopy of *Tg(actb2:H2B-2x-mScarlet;mpeg1.1:EMTB-3xGFP)* skin explants. White dotted outline indicates epidermal scratch. Yellow dotted line box in A is magnified in A′ insets. Yellow arrowheads indicate MTOC, white arrows and yellow dotted outlines indicate Langerhans cell nucleus, double-headed arrow indicates dendrites extending beyond the obstacle nucleus (yellow asterisk). *n*=21 out of 25 (84%) of cells exhibit MTOC-first phenotype from three experiments. (B) Representative still images from time-lapse confocal microscopy of vehicle-treated *Tg(actb2:H2B-2x-mScarlet;mpeg1.1:EMTB-3xGFP)*^+^ Langerhans cells navigating around obstacle keratinocytes towards a wound. Yellow arrowheads denote the MTOC, double-headed arrow indicates dendrites extending beyond the obstacle nucleus (yellow asterisks). (C,D) Representative still images from time-lapse confocal microscopy of paclitaxel-treated *Tg(actb2:H2B-2x-mScarlet;mpeg1.1:EMTB-3xGFP)*^+^ Langerhans cells attempting to navigate around obstacle keratinocytes towards a wound. C represents an unsuccessful navigation attempt; D represents a successful navigation. Yellow asterisks denote obstacle keratinocyte, yellow arrowheads denote MTOC, double-headed arrows indicate dendrites extending beyond the obstacle nucleus. (E) Quantification of navigation attempts in vehicle-treated and paclitaxel-treated conditions, *n*=38 attempts tracked from four experiments in vehicle conditions, *n*=31 attempts tracked from three experiments in paclitaxel conditions. (F) Violin plots showing the length of time required to navigate around keratinocyte, *n*=26 cells tracked from four experiments in vehicle conditions, *n*=6 cells tracked from three experiments in paclitaxel conditions. (G,H) Representative still images from time-lapse confocal microscopy of *Tg(actb2:H2B-2x-mScarlet;mpeg1.1:YFP)* skin explants treated with vehicle (G) or paclitaxel (H). White dotted lines indicates wound, arrows indicate phagocytic cells in wound margin, arrowheads indicate cells with stretched dendrites. (I) Violin plots showing the percentage of cells exhibiting phagocytosis or stretching after 2 h, *n=*299 cells counted from ten scales in vehicle conditions from four experiments, *n=*128 cells from 13 scales from three experiments. (J) Stepwise schematic showing a Langerhans cell (cyan) successfully navigating around an obstacle keratinocyte (asterisk). Fisher's exact test was used to determine significance in E by using the raw numbers of successful and unsuccessful navigations. Mann–Whitney *U*-test was used to determine significance in F. One-way ANOVA followed by Bonferroni post-test was used to determine significance in I. ns, not significant; **P<0.01, ****P<0.0001. Timestamps denote min:s post-scratch. Scale bars: 10 μm (A′–D), 100 μm (A,G,H).

*In vitro* studies using microfabricated constraints implicate microtubules and the MTOC in dictating polarity and decision-making in migrating immune cells (Kopf et al., 2020; Kroll et al., 2023; Renkawitz et al., 2019). This is accomplished by positioning the MTOC and microtubule-associated proteins either ahead of or behind the nucleus. Certain cell types, such as dendritic cells, often exhibit a nucleus-first mode of migration, whereas other cell types, such as neutrophils, can exhibit either MTOC- or nucleus-first migration (Etienne-Manneville, 2013). Therefore, we asked how the Langerhans cell MTOC was positioned relative to the nucleus during directional migration and whether or not its position corresponded to pathfinding decisions. By scratching skin explants from *Tg(mpeg1.1:EMTB-3xGFP;actb2:H2B-2x-mScarlet)* fish, we tracked the Langerhans cell MTOC and nucleus as the cell migrated towards the wound (Fig. 7A; Movie 7). We observed that the MTOC preceded the nucleus past the obstacle keratinocyte in 21/25 instances (Fig. 7A′).

Prior studies found that paclitaxel-mediated microtubule stabilization can inhibit cell migration (Ganguly et al., 2011; Wang et al., 2019) and centriole motility (Ching et al., 2022). To determine the effects of microtubule stabilization on Langerhans cell migration, we repeated the scratch injury assay in the presence of paclitaxel or vehicle control. Using skin explants from *Tg(mpeg1.1:EMTB-3xGFP;actb2:H2B-2x-mScarlet)* fish and observing MTOC position as a proxy for successful navigation, we examined how cells and microtubules behaved after paclitaxel treatment. We observed that 76% of vehicle-treated Langerhans cells successfully navigated past obstacle cells with an average navigation time of 675 s (Fig. 7B,E,F; Movie 8). By contrast, 77% of paclitaxel-treated Langerhans cells failed obstacle navigation (Fig. 7C–E). When paclitaxel-treated cells managed to successfully navigate past their obstacle, navigation times were significantly longer compared to those of control cells, averaging 2175 s (Fig. 7F). To observe possible outcomes from this inability to navigate the epidermis, we tracked Langerhans cell behaviors as they attempted to enter the wound margin. After 2 h in control conditions, we observed that a subset of Langerhans cells had entered the closing wound and engulfed debris [Fig. 7G, arrows; Fig. 7I, average of 20% of cells per wound margin region of interest (ROI)]. Less frequently, we noted Langerhans cells that did not enter the recovering wound but instead extended an elongated dendrite into the region (Fig. 7G, arrowhead; Fig. 7I, average of 4.7% of cells per wound margin ROI). In paclitaxel-treated conditions, Langerhans cells entered the wound margin to engulf debris at a significantly lower rate, and we observed a moderate increase in the number of cells that probed the wound with elongated dendrites (Fig. 7G, arrowhead; Fig. 7I). Taken together, these results suggest that Langerhans cells use MTOC positioning to aid in circumventing cellular obstacles during migration (Fig. 7J) and that stabilizing microtubules inhibits navigational pathfinding to wounds.

## DISCUSSION

How immune cells dynamically and rapidly navigate densely packed epithelia to respond to injury remains largely unknown. Here, we use scale explants from adult zebrafish skin as a tractable model to examine tissue-resident macrophage behaviors within a native epithelial microenvironment and apply acute chemical perturbations to modulate the microtubule cytoskeleton. We show that microtubule depolymerization alters Langerhans cell morphology, including dendrite number and length. We find that both the cell body and MTOC rapidly move towards the phagosome during debris engulfment and that microtubules are partially required for efficient debris engulfment. What functions might the microtubule cytoskeleton play during phagocytosis? Previous work using cultured macrophages found that the MTOC reorients during phagocytosis of antibody-coated beads and red blood cells and that microtubules were strictly required for engulfment (Athlin et al., 1986; Eng et al., 2007; Walter et al., 1980). Eng et al. (2007) posited that the MTOC can increase interactions between the Golgi complex and the phagosome, which could facilitate subsequent cross-presentation events between the phagocyte and other immune cells. By contrast, Möller et al. (2022) found that depolymerization of microtubules changed how zebrafish microglia encounter and engulf apoptotic corpses *in vivo*. In the absence of microtubules, microglia relied more heavily on a motility-based engulfment strategy (Möller et al., 2022), similar to our own observations (Fig. 3H–J), highlighting the flexible engulfment abilities of phagocytes within native microenvironments.

During tissue-level navigation through the epidermis to scratch wounds, we found that Langerhans cells frequently paused at sites of keratinocyte contact, which present potential decision points. These decision points are akin to microfluidic mazes developed to study immune cell migration decisions *in vitro* (Ambravaneswaran et al., 2010; Hadjitheodorou et al., 2023; Kopf et al., 2020; Kroll et al., 2023; Renkawitz et al., 2018; Tweedy et al., 2020). Whereas these microfluidic-based studies extensively probed how MTOC positioning influences navigation *in vitro*, our observations provide evidence that MTOC positioning provides assistance in navigation decision-making within an epithelial tissue. Our work found that microtubule stabilization via paclitaxel treatment prevents directional navigation around obstacle keratinocytes (Fig. 7B–F). However, our data cannot differentiate whether this is due to altering the microtubule cytoskeleton or preventing MTOC motility around the obstacle. Ultimately, cell-autonomous manipulations of the microtubule cytoskeleton, ideally using recently developed inducible protein degradation systems or optogenetic approaches (Borowiak et al., 2015; Boswell et al., 2025; Meiring et al., 2022; Yamaguchi et al., 2019), will allow us to distinguish between these possibilities.

Our perturbations of microtubule polymerization and stability affected Langerhans cell migration efficiency and actin localization. Our data suggest two non-mutually exclusive models in which microtubules and the MTOC promote efficient tissue-level migration: by decision-making during directional migration around obstacles and/or by regulating actin polarity. An important next step in dissecting this latter mechanism will be the development of methodology to specifically assess and manipulate GEF-H1 localization and RhoA activity in Langerhans cells.

Collectively, our work demonstrates requirements for microtubules in steady-state organ surveillance and directed cell migration by tissue-resident macrophages during wound repair. Tissue-level navigation by immune cells requires rapid navigational decision-making to mount a quick and efficient immune response. Our work highlights the challenges that immune cells face during directional navigation through an epithelium towards a stimulus and presents itself as a unique model for further study of how cells integrate the extracellular environment into decision-making. Uncovering the details and regulation of Langerhans cell motility are important not only for understanding responses to tissue injury, but also for how these cells initially seed the skin and emigrate from the skin to secondary immune organs.

## MATERIALS AND METHODS

### Key resource table

Information on animal strains used, chemicals, antibodies, recombinant DNA and software can be found in  Table S1.

### Zebrafish husbandry

Zebrafish were housed at 26–27°C on a 14/10 h light cycle. The strains used are listed in Table S1. Animals aged 6–18 months of either sex were used in this study. All zebrafish experiments were approved by the Institutional Animal Care and Use Committee at the University of Washington (Protocol #4439-01).

### Generation of transgenic zebrafish

To generate *Tg(mpeg1.1:EMTB-3xGFP)^w270Tg^*, a previously published *mpeg1.1:EMTB-3xGFP* plasmid (Barros-Becker et al., 2017) and *tol2* mRNA were injected into AB embryos at the one-cell stage, and embryos were raised to adulthood. Adults were screened for GFP^+^ cells in the skin, and GFP^+^ adults were then outcrossed to wild-type partners. F1 fish were raised to adulthood, when GFP expression was assessed in Langerhans cells to identify a line to propagate for this study.

To generate *Tg(mpeg1.1:EB3-GFP)* G0 animals, our published *tol2* plasmid containing *tol2* sites, the *mpeg1.1* promoter and the *cryaa:DsRed* transgenesis marker (Peterman et al., 2024) was modified to insert EB3-GFP downstream of the *mpeg1.1* promoter. To amplify EB3-GFP, Addgene plasmid #190164 (RRID:Addgene_190164, deposited by Anna Akhmanova) was used as template and amplified with the primers EB3-F and EB3-R. Our previously published *mpeg1.1* promoter-containing plasmid was amplified using the primers mpeg1.1_V_F and mpeg1.1_V_R. Gibson assembly was used to construct the final pTol2-*mpeg1.1:EB3-GFP;cryaa:DsRed* plasmid. *tol2* mRNA and pTol2-*mpeg1.1:EB3-GFP;cryaa:DsRed* plasmid DNA were injected into *Tg(mpeg1.1:mCherry)* embryos at the one-cell stage, and larvae were screened for *cryaa:DsRed* expression. Positive animals were raised to adulthood, when GFP expression was assessed in Langerhans cells.

To generate *Tg(actb2:h2b-mscarlet-mscarlet)^hm63Tg^* animals, Gibson assembly was used to create an H2B-2x-mScarlet fusion protein by adding a linker between two copies of mScarlet and fusing this to the C-terminus of human histone 2B. This was cloned into the pMTB expression construct that contains the zebrafish *actb2* enhancer/promoter inside a mini-Tol2 transposon system (Xiong et al., 2013), which was used for generating stable transgenics. *tol2* mRNA and plasmid DNA were injected into AB embryos at the one-cell stage. Potential founders were screened for bright red ubiquitous fluorescence and outcrossed to obtain a stable line.

### Scale removal

For scale removal, adult fish were anesthetized in system water containing 200 µg ml^−1^ buffered tricaine. Individual scales were removed with forceps, placed onto 6 mm plastic dishes, epidermis side up, and allowed to adhere for 30 s before adding L-15 medium prewarmed to room temperature (22°C). Following scale removal, animals were recovered in system water.

### Scale injury assay

For the epidermal scratch assay (Figs 4–7), one pair of forceps was used to assist in pinning the scale down by contacting a region devoid of epidermis on the anterior scale surface. A second pair introduced the scratch in the middle of the epidermis. Scratches were only used for data collection if they did not extend to the edge of the scale and were oval in shape (as shown by representative images in Fig. 3A,B). When treating scales with pharmacological agents, scales were removed, placed in L-15 medium, and pretreated with vehicle control or pharmacological agent. Nocodazole-treated (50 µM) scales were pretreated for 10 min. Paclitaxel-treated (25 µM) scales were pretreated for 30 min. After preincubation, scales were placed under a dissecting microscope, scratched and placed under the confocal microscope for imaging.

For the dual nocodazole+ROCK inhibitor treatments, scales were first treated with Y-27632 (40 min, 25 µM) followed by nocodazole treatment (10 min, 50 µM). After the 10 min incubation with nocodazole, scales were placed under a dissecting microscope, scratched and placed under the confocal microscope for imaging.

### Microscopy and live imaging

An upright Nikon Ni-E microscope equipped with an A1R MP+ confocal scanner, a piezo *z*-drive (Mad City Labs Nano-F450) and a 25× water dipping objective (1.1 NA; Nikon MRD77220), was used for all experiments unless noted elsewhere. Laser powers between 0.5 and 1% were used for 488 and 561 nm lasers in conjunction with 521/42 nm and 600/45 nm emission filter sets for GFP and RFP channels, respectively. *Z*-stacks were acquired using a resonant scanner and post-processed to remove noise using the denoise.ai function in NIS-Elements.

Super-resolution imaging was performed using a Zeiss LSM 980 with Airyscan 2 in SR-4y mode, as previously described (Hewitt et al., 2024). A Zeiss LD LCI Plan-Apochromat 40×/1.2W numerical water objective was used. Airyscan Processing was applied to all raw images using the 3D default settings for pixel reassignment and deconvolution using Zen Blue software 3.7.9.

### Cell staining

#### Live-cell staining with BodipyTR

Whole fish were incubated in BodipyTR (stock solution 0.5 mM) at a dilution of 1:20,000 for 3 days. Scales were removed and imaged as described above.

#### Fixed cell staining

Scales from *Tg(mpeg1.1:mCherry;mpeg1.1:EMTB-3xGFP)* fish were removed and immediately placed into 4% paraformaldehyde diluted in 1× phosphate buffered saline. Scales were fixed for 20 min at room temperature. Scales were washed with 1× phosphate buffered saline four times for 10 min per wash. Following the fourth wash, scales were incubated for 2 h at room temperature in 10% fetal bovine serum and 0.2% Tween 20 diluted in 1× phosphate buffered saline. Following block and permeabilization, scales were stained with a mouse anti-γ-tubulin antibody at a dilution of 1:2000 and a chicken anti-GFP antibody at a dilution of 1:500 overnight on a nutator at 4°C. After overnight staining, scales were washed with 1× phosphate buffered saline four times for 15 min per wash. Following the fourth wash, scales were stained with a goat anti-mouse Alexa Fluor 647 secondary antibody at a dilution of 1:500 and a goat anti-chicken Alexa Fluor 488 secondary antibody at a dilution of 1:1000 for 2 h on a nutator at room temperature. Following secondary staining, scales were washed with 1× phosphate buffered saline four times for 10 min per wash. Scales were mounted on glass slides in ProLong Gold. Scales were imaged on the Nikon Ni-E microscope described above using a 40× (NA1.3) oil immersion objective. See Table S1 for details on antibodies used.

### Steady-state chemical treatments

For nocodazole and paclitaxel treatments, scales were removed, placed onto dry 6 mm dishes and incubated in 5 ml L-15 medium. Imaging had commenced for at least 10 min before careful addition of chemicals while on the microscope stage. Appropriate vehicle controls (DMSO) were used at equivalent %v/v.

### Laser-induced cell damage

For laser-induced cell damage, scales were mounted as described above. Target cells at least one cell distance away from a Langerhans cell (∼5–15 μm) and within the same *z*-plane were located and ablated using a UGA-42 Caliburn pulsed 532 nm laser (Rapp OptoElectronic). The laser was focused through a 25× objective at 4× zoom. Ablation was produced in the focal plane using 15–20% power at a single point within a nucleus, firing three times for 3 s each using a custom NIS-Elements macro. In the presence of nocodazole (Fig. 3), scales were first pretreated with nocodazole (50 µM) for 10 min, followed by laser-induced cell damage and subsequent confocal imaging.

### Image analysis

All image analyses were performed in Fiji/ImageJ (Schindelin et al., 2012).

#### Dendrite metrics

Dendrites were counted as microtubule positive if the ratio of EMTB-3xGFP/mCherry in the dendrite was greater than 0.5. To quantify dendrite number and length in Fig. 1, dendrites were manually counted and measured every 10 min throughout the treatment time course. Numbers and lengths across the time course were averaged to obtain values shown in Fig. 1.

#### Sholl analysis

To calculate Sholl crossings, five timepoints from a time-lapse micrograph were selected for each cell. Each cell was thresholded and the Sholl Analysis Function in the Neuroanatomy plugin in ImageJ was used to calculate Sholl crossings (Arshadi et al., 2021; Ferreira et al., 2014). The resulting visualization was manually inspected to ensure accuracy. The sum of the radii crossings for each cell across the time points was calculated, and the percentage of total crossings was summed for each condition. The average crossings per condition amongst cells was then computed and graphed.

#### Cell motility

To calculate cell motility metrics (displacement, total distance traveled, meandering index) in Fig. 4, cells were thresholded and tracked using the LAP algorithm in ImageJ (Ershov et al., 2022; Tinevez et al., 2017). Cells were only tracked and quantified if they could be tracked throughout the entire time course. Cells were excluded if their displacement was less than 10 μm. To quantify the meandering index for each individual cell, the displacement of that cell was divided by its total distance traveled. To calculate cell distance from phagosome in Fig. 3E, cells were thresholded and tracked using the LAP algorithm in ImageJ. *xy* coordinates of the tracked cell were used to calculate distance from phagosome at each time point.

To calculate the cell number in wound margin in Fig. 4F, a rectangular region of 150 μm wide and (length of wound×1.2) μm long was drawn around the wound. The number of cells was counted for each timepoint and normalized to time 0. Cells were only counted if >50% of their cell body was within the ROI. To calculate the engulfing and stretching phenotypes in Fig. 7, the total number of cells at 2 h within the wound margin [rectangular region of 150 μm wide and (length of wound×1.2) μm long] was counted. A second region was drawn around the closing wound (as seen in Fig. 7G,H). Cells were scored as engulfing if they were within the wound and presenting a rounded, phagocytic phenotype. Cells were scored as stretching if they extended dendrites, but not the cell body, into the wound. Frames prior to the 2 h time were used as references to score if cells were morphologically changing into either of these phenotypes.

#### Trailing and leading edge Lifeact intensity

To calculate the trailing/leading edge Lifeact-mRuby ratio in Fig. 5, a set of custom ImageJ macros were used. Migrating cells were thresholded and outlined over time. The outlines were used as ROIs, bisected to make a trailing half and leading half, and mean Lifeact-mRuby intensity in each half was quantified at each time point. Last, the mean of means for trailing and leading halves over the course of migration was calculated. The ratio of trailing/leading means for the course of migration for each cell was plotted.

#### MTOC motility and positioning

MTOCs were manually tracked in ImageJ in order to quantify their speed and distance from phagosome (Fig. 3A–G). To score MTOC decision-making in Fig. 7A, a decision was scored as MTOC-first if the MTOC was the first structure to move past the obstacle nucleus. A decision was scored nucleus-first if the nucleus preceded the MTOC. To score successful navigation and navigation times in Fig. 7B–F, the difference in time between an MTOC first encountering a nucleus and its successful progression past the obstacle was calculated. Navigations were scored unsuccessful if the MTOC did not progress past the obstacle nucleus within at least a 55 min observation window.

#### Wound area quantification

To calculate wound area, an ROI was drawn manually around the wound at time 0, and at each hour post-wounding up to 4 h. Areas were normalized to the size of the wound at time 0.

### Statistical analysis

GraphPad Prism was used to generate graphs and perform statistical analyses. At least three individual biological experiments were performed unless otherwise noted. Tests used, number scales, cells and ROIs are described in figure legends.

## Supplementary Material

10.1242/joces.264101_sup1 Supplementary information
