# Supplementary material for: Microtubules regulate tissue-level navigation in skin-resident macrophages
Source: J Cell Sci. 2025 Sep 18;138(18):jcs264101. doi: 10.1242/jcs.264101 (PMC12516128; doi:10.1242/jcs.264101)
Supplement: Supplementary information [file joces-138-264101-s1.pdf]

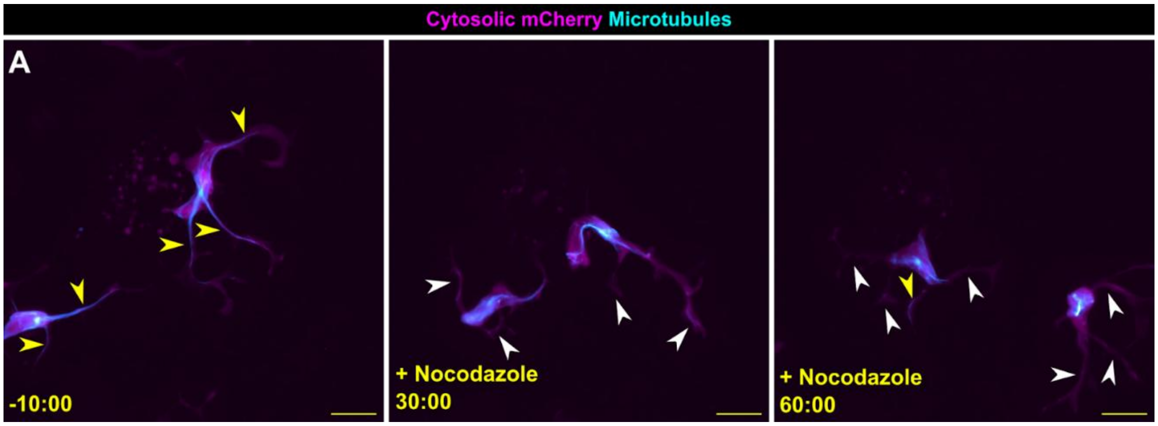


**Fig. S1. Confirmation of the EMTB-3xGFP reporter for tracking microtubules. A.** Representative images from confocal time lapse microscopy of *Tg(mpeg1.1:mCherry;mpeg1.1:EMTB-3xGFP)*+ Langerhans cells showing effects of nocodazole treatment on EMTB signal within Langerhans cells. Yellow arrowheads indicate EMTB-positive dendrites, white arrowheads indicate EMTB-negative dendrites. Timestamps denote mm:ss relative to the addition of nocodazole. Scale bar, 10 µm.

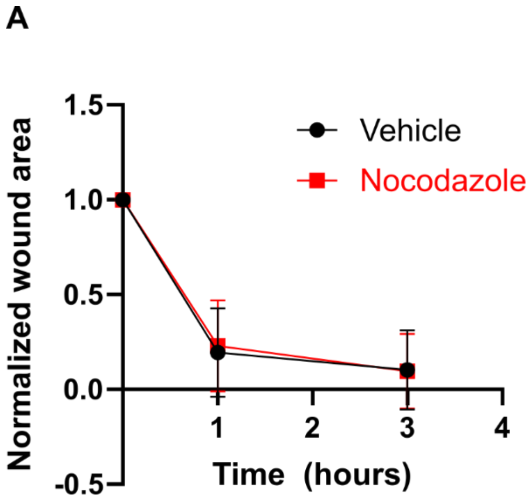


**Fig. S2. Nocodazole treatment does not impact wound closure. A.** Quantification of wound closure area in the presence of nocodazole or vehicle control, *n* = 13 skin explants from 3 experiments for vehicle conditions, *n* = 9 skin explants from 3 experiments for nocodazole conditions. Dots represent averages, error bars represent standard deviation. Two-way ANOVA followed by Bonferroni post-tests revealed no significant differences ($p > 0.05$).

Journal of Cell Science • Supplementary information

**Table S1. Key Resources Table**

| **Reagent or Resource** | **Source** | **Identifier** |
|---|---|---|
| **Experimental models: Organisms** | | |
| Zebrafish: AB | This paper | ZFIN: ZDB-GENO-960809-7 |
| Zebrafish: *Tg(mpeg1.1:mCherry)$^{gl23Tg}$* | (Ellett et al., 2011) | ZFIN: ZDB-TGCONSTRCT-120117-2 |
| Zebrafish: *Tg(mpeg1.1:YFP)$^{w200Tg}$* | (Roca and Ramakrishnan, 2013) | ZFIN: ZDB-TGCONSTRCT-130130-3 |
| Zebrafish: *Tg(actb2:h2b-mscarlet-mscarlet)$^{hm63Tg}$ (referred to as Tg(actb2:H2B-2x-mScarlet))* | This paper | n/a |
| Zebrafish: *Tg(mpeg1.1:EMTB-3xGFP)$^{w270Tg}$* | This paper | n/a |
| Zebrafish: *Gt(ctnna1-Citrine)$^{ct3aGt}$* | (Trinh et al., 2011) | ZFIN: ZDB-ALT-111010-23 |
| Zebrafish: *Tg(mpeg1.1:Lifeact-mRuby)$^{w269Tg}$* | (Peterman et al., 2024) | n/a |
| **Chemicals, peptides, and recombinant proteins** | | |
| L-15 media | Gibco | 21083027 |
| Ethyl 3-aminobenzoate methanesulfonate | Millipore Sigma | E10521 |
| DMSO | Acros Organics | 295522500 |
| Nocodazole | Millipore Sigma | M1404 |

Journal of Cell Science • Supplementary information

| | | |
|---|---|---|
| Y-27632 2HCL | SelleckChem | S1049 |
| Paclitaxel | Cayman Chemical | 10461 |
| BodipyTR-BSA | Invitrogen | B34400 |
| ProLong Gold | Invitrogen | P36930 |
| Fetal Bovine Serum | VWR | 89510-182 |
| Tween20 | Promega | H5152 |
| Paraformaldehyde | Electron Microscopy Sciences | 15710 |
| **Antibodies** | | |
| Chicken anti-GFP | Genetex | Cat# GTX13970, RRID:AB_371416 |
| Mouse anti-γ-tubulin | Sigma | Cat# T6557, RRID:AB_477584 |
| Goat anti-chicken AlexaFluor 488 | Invitrogen | Cat# A11039, RRID:AB_142924 |
| Goat anti-mouse AlexaFluor 647 | Invitrogen | Cat# A32728, RRID:AB_2633277 |
| **Recombinant DNA** | | |
| *mpeg1.1:EMTB-3xGFP* | (Barros-Becker et al., 2017) | n/a |
| *mpeg1.1:EB3-GFP* | This paper | n/a |
| EB3-F 5' GTGCAGGAGACAGCAAAACAGCC ACCATGGCCGT 3 | This paper | n/a |

| | | |
|---|---|---|
| EB3-R 5' TAGTTCTAGAGGCTCGAGAGTTAC TTGTACAGCTCGTCCATGCCGAG AGTG 3' | This paper | n/a |
| mpeg1.1_V_F 5' TGGACGAGCTGTACAAGTAACTCT CGAGCCTCTAGAACTATAGTGAGT 3' | This paper | n/a |
| mpeg1.1_V_R 5' ACATTGACGGCCATGGTGGCTGT TTTGCTGTCTCCTGCACTAATG 3' | This paper | n/a |
| **Software and algorithms** | | |
| Fiji/ImageJ | (Schindelin et al., 2012) | RRID:SCR_002285 |
| NIS-Elements 5.30 | Nikon | RRID:SCR_014329 |
| Zen Blue 3.7.9 | Zeiss | RRID:SCR_013672 |
| Prism 9 | GraphPad | RRID:SCR_002798 |

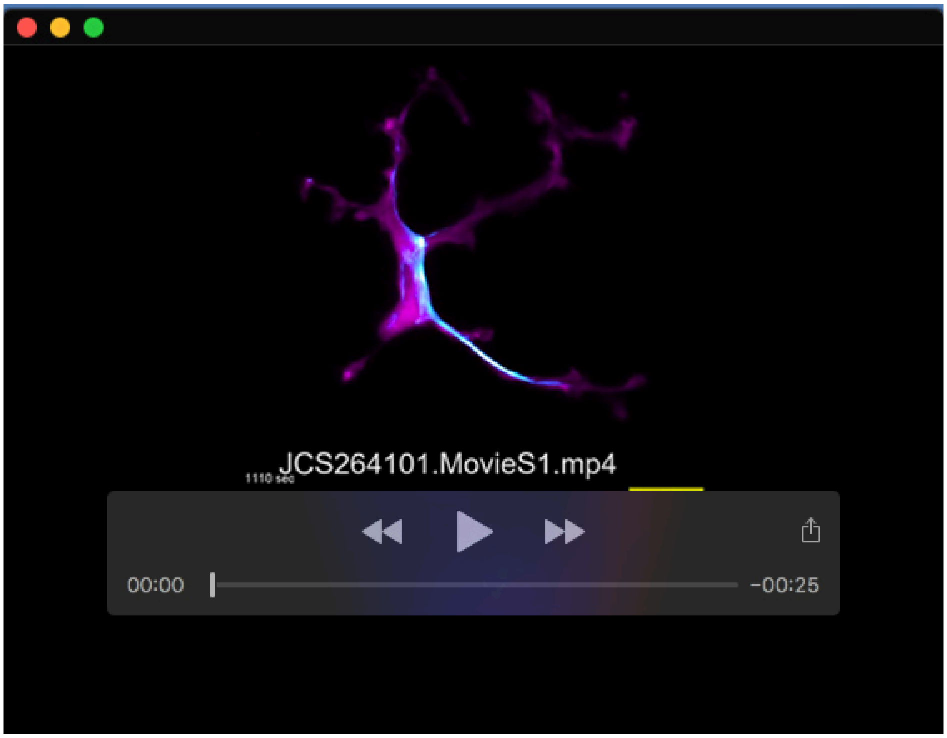


**Movie 1.** Time-lapse microscopy of Langerhans cell (magenta; *Tg(mpeg1.1:mCherry)*) expressing the microtubule reporter EMTB-3xGFP (cyan; *Tg(mpeg1.1:EMTB-3xGFP)*). Scale bar, 10 µm.

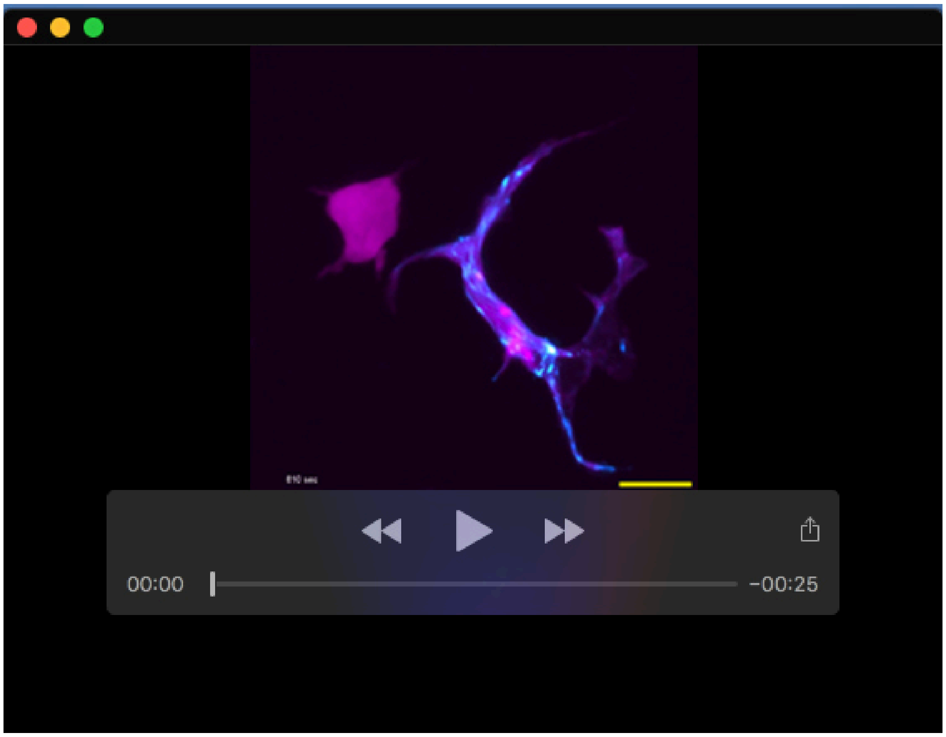


**Movie 2.** Time-lapse microscopy of Langerhans cell (magenta; *Tg(mpeg1.1:mCherry)*) expressing the plus-end microtubule reporter EB3-GFP (cyan). Scale bar, 10 µm.

Journal of Cell Science • Supplementary information

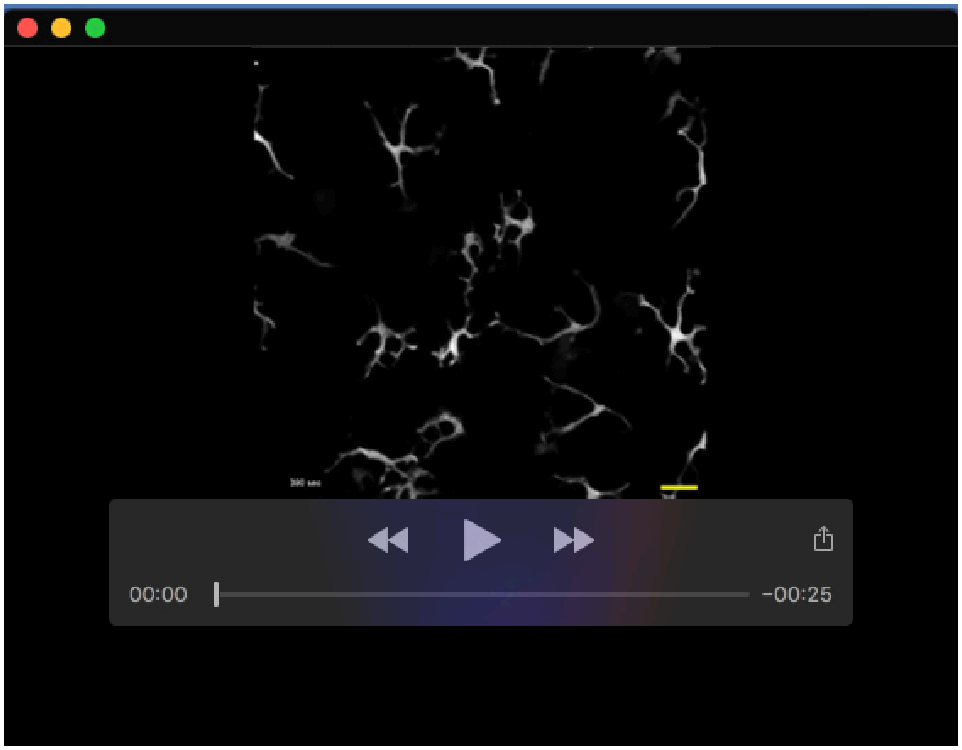


**Movie 3.** Time-lapse microscopy of Langerhans cells (white; *Tg(mpeg1.1:YFP)*) treated with vehicle, nocodazole, or paclitaxel in steady-state conditions. Scale bar, 20 μm.

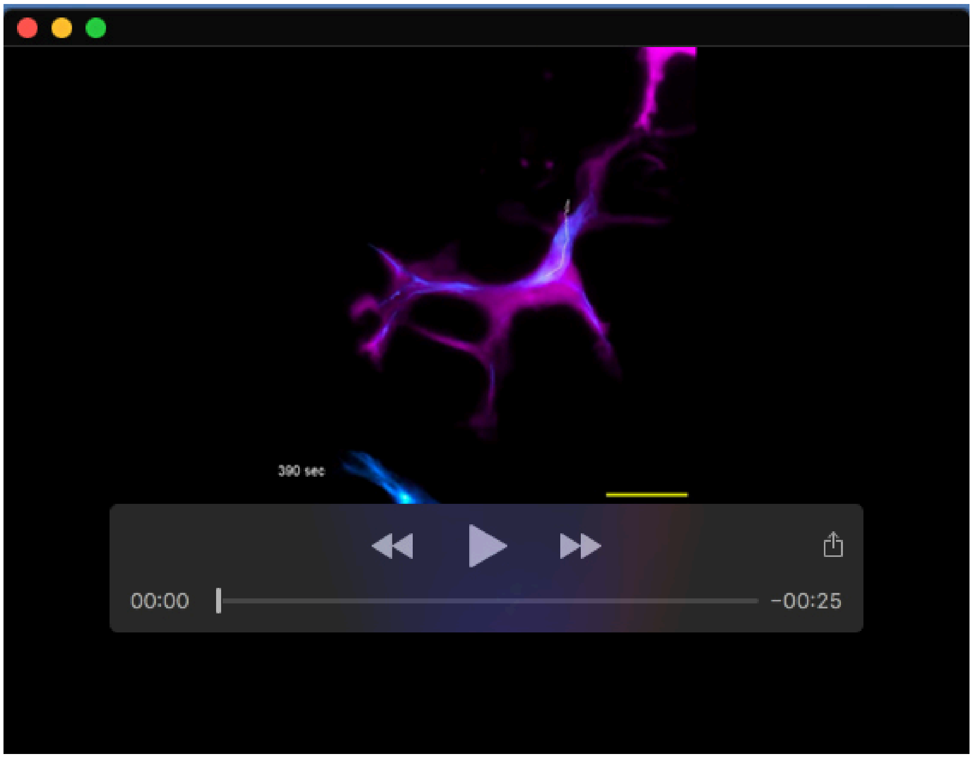


**Movie 4.** Time-lapse microscopy of a Langerhans cell (magenta; *Tg(mpeg1.1:mCherry)*) MTOC during debris engulfing via tracking of EMTB-3xGFP foci (*Tg(mpeg1.1:EMTB-3xGFP)*). Asterisk indicates site of keratinocyte laser ablation, white line traces the MTOC motility during engulfment. Scale bar, 10 μm.

Journal of Cell Science • Supplementary information

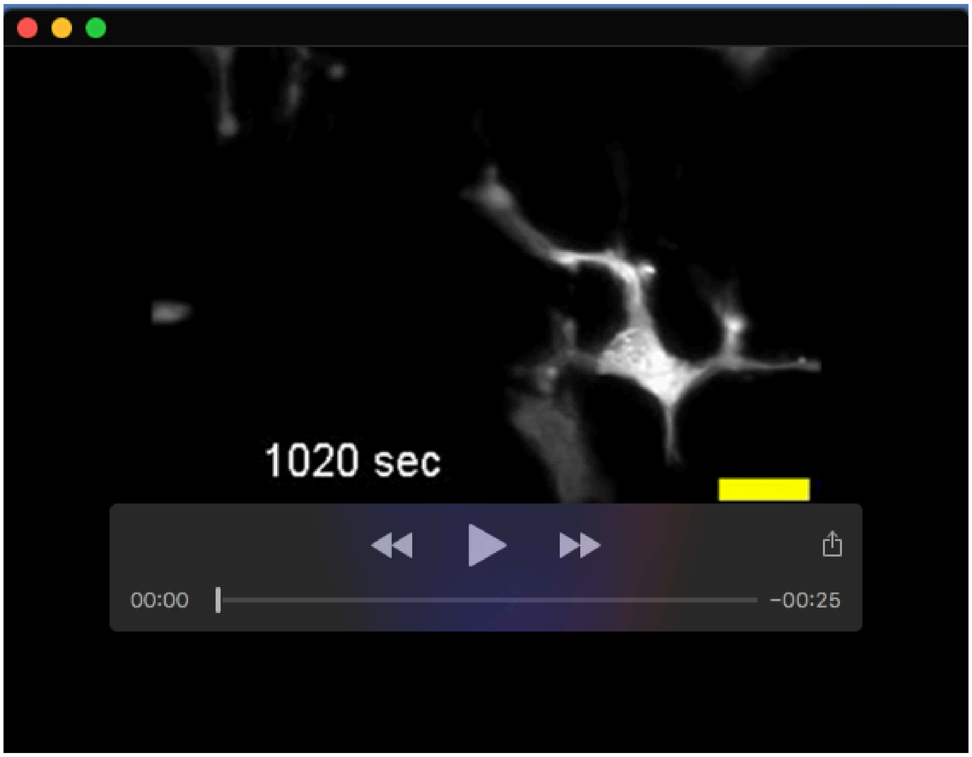


**Movie 5.** Time-lapse microscopy of Langerhans cell (white; *Tg(mpeg1.1:YFP)*) engulfing cellular debris generated after laser-induced damage of keratinocytes. Cells are treated with vehicle or nocodazole. Asterisk indicates site of keratinocyte laser ablation. Scale bar, 10 μm.

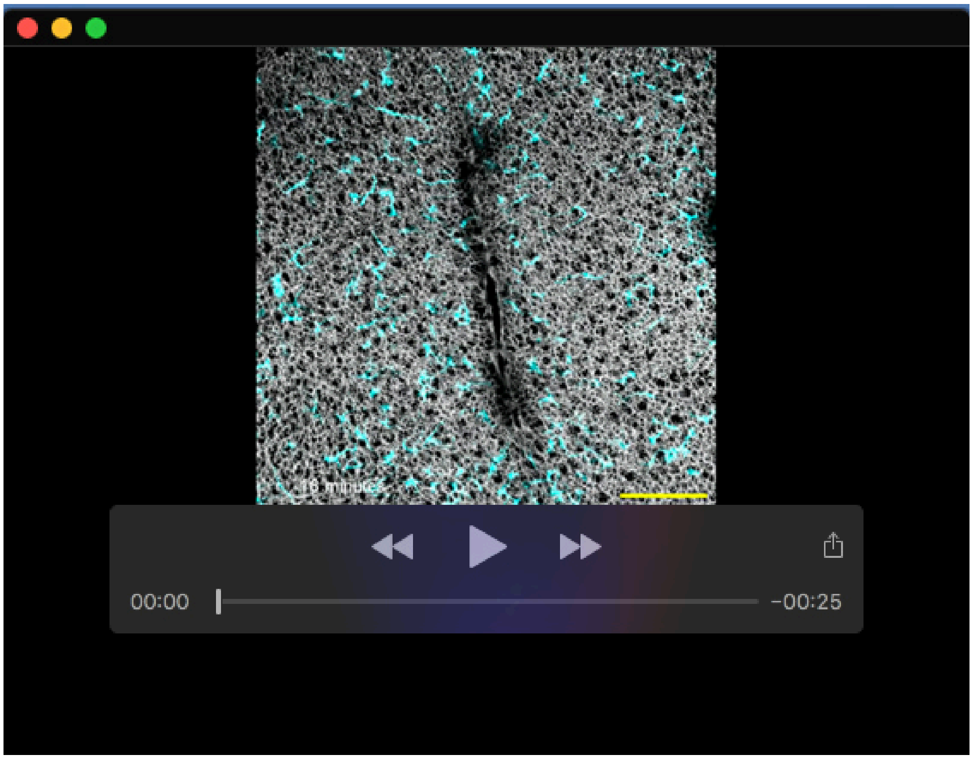


**Movie 6.** Time-lapse microscopy of Langerhans cells (cyan; *Tg(mpeg1.1:mCherry)*) reacting to epidermal wounds (epidermal cells labeled in white; *Gt(ctnna1-Citrine)*). Cells are treated with vehicle or nocodazole. Scale bar, 100 μm.

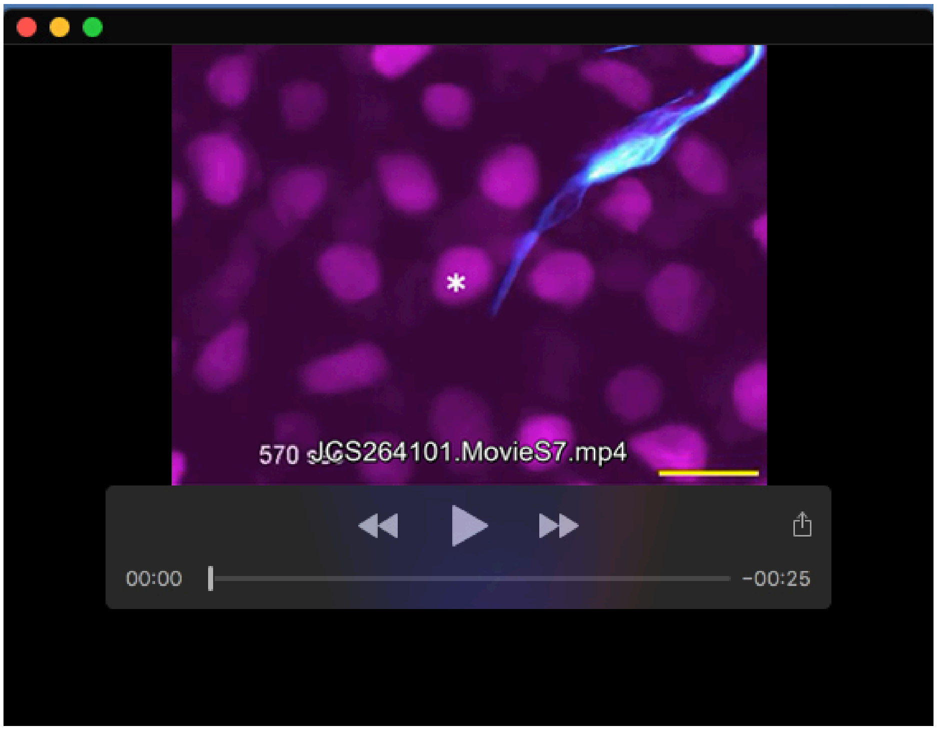


**Movie 7.** Time-lapse microscopy of MTOC-labeled Langerhans cell (cyan; *Tg(mpeg1.1:EMTB-3xGFP)*) navigating around obstacle nuclei (magenta;*Tg(actb2:H2B-2x-mScarlet)*) in order to reach the epidermal wound. Asterisk indicates an obstacle nucleus. Scale bar, 10 µm.

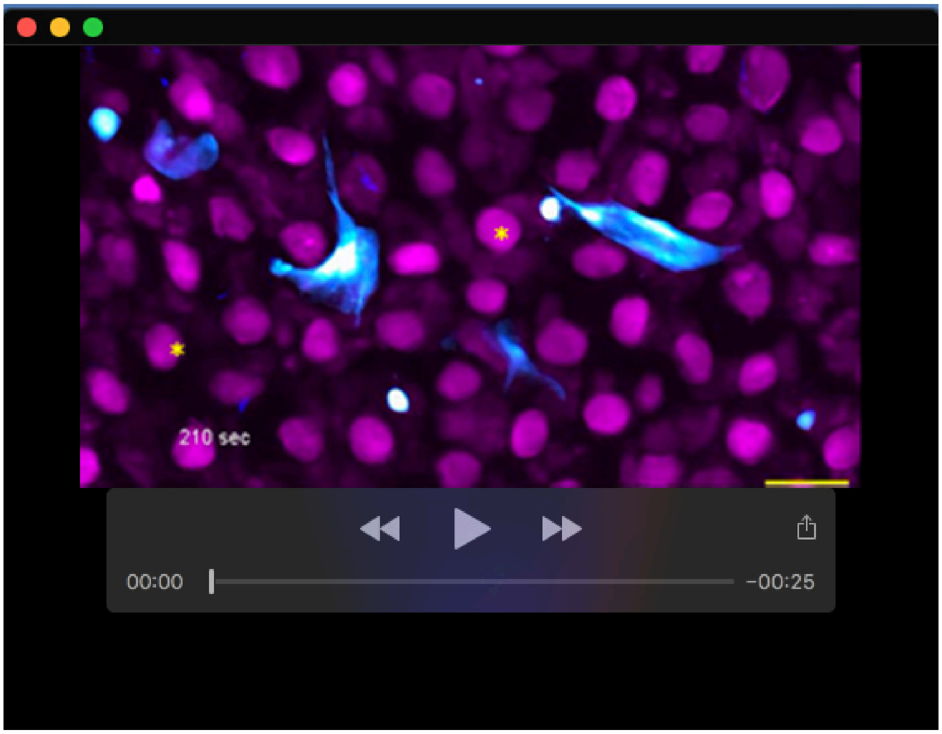


**Movie 8.** Time-lapse microscopy of MTOC-labeled Langerhans cell (cyan;*Tg(mpeg1.1:EMTB-3xGFP)*) navigating around obstacle nuclei (magenta;*Tg(actb2:H2B-2x-mScarlet)*) in order to reach the epidermal wound. Cells are treated with vehicle or paclitaxel. Asterisk indicates an obstacle nucleus. Scale bar, 10 µm.
